# Exposure to Vicarious Social Defeat Stress and Western-Style Diets During Adolescence Leads to Physiological Dysregulation, Decreases in Reward Sensitivity, and Reduced Antidepressant Efficacy in Adulthood

**DOI:** 10.3389/fnins.2021.701919

**Published:** 2021-08-02

**Authors:** Omar K. Sial, Tamara Gnecco, Astrid M. Cardona-Acosta, Emily Vieregg, Ernesto A. Cardoso, Lyonna F. Parise, Carlos A. Bolaños-Guzmán

**Affiliations:** ^1^Department of Psychological and Brain Sciences, and Institute for Neuroscience, Texas A&M University, College Station, TX, United States; ^2^Fishberg Department of Neuroscience, Icahn School of Medicine at Mount Sinai, New York, NY, United States

**Keywords:** adolescence, early-life stress, western-style, high-fat diet, depression, antidepressant efficacy

## Abstract

A dramatic increase in the prevalence of major depression and diet-related disorders in adolescents has been observed over several decades, yet the mechanisms underlying this comorbidity have only recently begun to be elucidated. Exposure to western-style diet (WSD), high in both fats (45% kcal) and carbohydrates (35% kcal): e.g., high fat diet (HFD), has been linked to the development of metabolic syndrome-like symptoms and behavioral dysregulation in rodents, as similarly observed in the human condition. Because adolescence is a developmental period highlighted by vulnerability to both stress and poor diet, understanding the mechanism(s) underlying the combined negative effects of WSDs and stress on mood and reward regulation is critical. To this end, adolescent male C57 mice were exposed to vicarious social defeat stress (VSDS), a stress paradigm capable of separating physical (PS) versus psychological/emotional (ES) stress, followed by normal chow (NC), HFD, or a separate control diet high in carbohydrates (same sucrose content as HFD) and low in fat (LFD), while measuring body weight and food intake. Non-stressed control mice exposed to 5 weeks of NC or HFD showed no significant differences in body weight or social interaction. Mice exposed to VSDS (both ES and PS) gain weight rapidly 1 week after initiation of HFD, with the ES-exposed mice showing significantly higher weight gain as compared to the HFD-exposed control mice. These mice also exhibited a reduction in saccharin preference, indicative of anhedonic-like behavior. To further delineate whether high fat was the major contributing factor to these deficits, LFD was introduced. The mice in the VSDS + HFD gained weight more rapidly than the VSDS + LFD group, and though the LFD-exposed mice did not gain weight as rapidly as the HFD-exposed mice, both the VSDS + LFD- and VSDS + HFD-exposed mice exhibited attenuated response to the antidepressant fluoxetine. These data show that diets high in both fats and carbohydrates are responsible for rapid weight gain and reduced reward sensitivity; and that while consumption of diet high in carbohydrate and low in fat does not lead to rapid weight gain, both HFD and LFD exposure after stress leads to reduced responsiveness to antidepressant treatment.

## Introduction

The co-occurrence of mood disorders (e.g., major depressive disorder, generalized anxiety disorder, bipolar disorder, etc.) with diet-related diseases (e.g., metabolic syndrome [MetS], cardiovascular disease [CVD], etc.) has become a well-recognized phenomenon in recent years despite the underlying mechanisms being severely understudied (Pan et al., 2012; Tang et al., 2017). Obesity, which is an excess accumulation of adipose tissue, is a highly prevalent and precarious ailment that affects approximately 40% of US population (Hales et al., 2020). The danger of obesity lies in that it is a major risk factor for life-threatening disorders such as MetS, CVD, stroke, and cancer (Huang, 2009; American College of Cardiology/American Heart Association Task Force on Practice Guidelines, Obesity Expert Panel 2013, 2014). Adipose tissue itself is metabolically active and acts to regulate energy homeostasis (Friedman, 2014) and releases proinflammatory agents that have been associated with risk of mood disorders (Byrne et al., 2015). The rate of adolescent obesity has been steadily increasing and has been correlated with an increased risk for major depressive disorder (MDD), the leading cause of disability worldwide (Mannan et al., 2016; Friedrich, 2017). Obesity and other diet-related metabolic disorders influence the prognosis of MDD, as it accounts for a 4-fold increase in premature death in depressed patients (Marazziti et al., 2014). Conversely, the presence of early-life depression is associated with an increased risk for obesity later in life (Marmorstein et al., 2014). This paucity of research on the comorbidity is likely due to the behavioral and biological complexities of these individual disorders, the involvement of multiple biological systems and the interplay between the central and peripheral nervous system. Adolescence is a period of enhanced vulnerability to stress, which can precipitate the emergence of major depressive disorder (MDD) and anxiety-related disorders (Eiland and Romeo, 2013), often causing life-long detriments (Kessler et al., 2007; Merikangas et al., 2010). Afflicted youth often adopt poor eating habits (Muñoz et al., 1997; O’Neil et al., 2014; Peeters, 2018), deriving sources of energy from foods high in fats and sugar (Reedy and Krebs-Smith, 2010). MDD is highly comorbid with obesity which may be further precipitated by stress (van Reedt Dortland et al., 2010; Alastalo et al., 2013; Loria et al., 2014; Vogelzangs et al., 2014; Weder et al., 2014). This is concerning as there is a bidirectional relationship between MDD and obesity, with each predicting the onset of the other (van Reedt Dortland et al., 2010; Vogelzangs et al., 2014). The symptomatology of mood- and diet-related disorders often overlap and, though well-recognized, the mechanism(s) underlying this comorbidity is not well-understood (Mansur et al., 2015). However, there is a lack of translational research models that aim to understand how stress and diet interact simultaneously to precipitate symptoms like those present in various comorbid mood and diet-related disorders. Given the increased prevalence of both MDD (Mojtabai et al., 2016) and obesity (Weiss et al., 2004; Marmorstein et al., 2014) in adolescents over the years, it is crucial to address this issue early in life to harbor better functionality in these individuals as they progress into adulthood.

The consumption of poor diets is the largest modifiable risk factor of early death globally (GBD 2015 Risk Factors Collaborators, 2016; Reisch, 2016). The western-style diet (WSD) also referred to as western-pattern diet, contains large amounts of saturated fats, sugars and refined grains (Shakersain et al., 2016) has been associated with long-term impairments such as dyslipidemia (Asadi et al., 2020), chronic inflammation (Neustadt, 2006), and cognitive decline (Shakersain et al., 2016). Diet-induced obesity paradigms have commonly been used to model symptoms of MetS and CVD. Preclinical studies have used calorically dense WSDs such as those that are high in either fats and/or carbohydrates to resemble the average unhealthy diet. Most studies using WSDs to induce obesogenic phenotypes in adult rodents contain 60% kcal from fat, which is significantly higher than the typical human WSD (Speakman, 2019). Dietary reference intakes recommend that adolescents consume 25–35% of kcal from fats, 45–65% from carbohydrates, and 10–30% from protein. A western-style diet would exceed >∼35% from fat and/or >∼65% from carbohydrates (Institute of Medicine (US) Committee to Review Dietary Reference Intakes for Vitamin D and Calcium, 2011). Additionally, the role of fats in the manifestation of these disorders has been an area of intense research for several decades, whereas the contribution of carbohydrates has largely been minimized (Kearns et al., 2018). High carbohydrate diets and the consumption of sugary beverages also pose a threat as they are known to increase serum triglycerides, and reduction in beneficial high density lipoprotein cholesterol (Coulston et al., 1983). There is a lack of studies that differentiate the effects of macronutrient content on experimental endpoints. Rodent studies utilize these very-high fat WSDs to expedite physiological and behavioral deficits, however, it can still take 6–20 weeks of consumption for deficits to emerge, resulting in rodents tested in late-adulthood and, at times, bordering geriatric age time-points (Moreno-Fernández et al., 2018) - further amplifying the need for early-life models.

The role of stress is likely a central factor in modulating this comorbidity, however, it is vastly understudied. Various stress paradigms have shown to dysregulate metabolic parameters and alter physiological responses, though the results can vary greatly. Chronic stressors can results in increases (Pecoraro et al., 2004; Coccurello et al., 2018) or decreases (Rabasa et al., 2011; Wang et al., 2012) in food intake, increased (Farias-Silva et al., 2002) or reduced (Foster et al., 2006) insulin concentrations, and changes in glucose levels (Farias-Silva et al., 2002). Much of this variation may be due to the nature and magnitude of the stressor. The chronic social defeat stress (CSDS) paradigm is an ethologically relevant and robust stressor that produces metabolic abnormalities that could play a role in the pathogenesis of obesity, such as increases in proinflammatory cytokines (Hodes et al., 2014) as well as microbiota alterations (Aoki-Yoshida et al., 2016; Bharwani et al., 2016). When a type of WSD that is high in both fats and carbohydrates, referred to here as high-fat diet (HFD; 45% kcal fat, 35% kcal sucrose), is given after CSDS, mice show dysregulated body weight and lipid synthesis (Chuang et al., 2010), while other studies have shown HFD buffering against stress (Finger et al., 2011; MacKay et al., 2017). Most of these findings have been derived from adult rodents, forgoing adolescence, a developmental period in which most of these symptoms often emerge in humans (Kessler et al., 2007; Mojtabai et al., 2016). Furthermore, the prevalence of psychological/emotional stress in childhood is about four times that of physical stress (Vachon et al., 2015) and has been shown to be highly predictive of the development of mental disorders later in life (Kessler et al., 2010). Given that solely witnessing a traumatic event is sufficient to precipitate anxiety and depressive symptoms (Motta et al., 2004), it is problematic that most animal models are unable to distinguish between physical and psychological stressors. Vicarious social defeat stress (VSDS) is a modified version of CSDS where a C57BL/6J mouse solely witnesses a physical defeat for 10 days and thus experiences psychological/emotional stress (Sial et al., 2016). The emotionally stressed mice exhibit increases in corticosterone levels and depression- and anxiety-like behaviors similar to the physically stressed mice, despite never have been defeated physically (Warren et al., 2020). Interestingly, deficits in social interaction and sucrose preference are observed in the long-term, emphasizing the salient and robust effect of the emotional stressor (Warren et al., 2013). To our knowledge, the effect of early-life emotional stress and exposure to different WSDs has not yet been explored. To address this gap, we exposed adolescent mice (postnatal day; PD35) to the VSDS paradigm in conjunction with WSDs: high in both fats and carbohydrates (HFD), or low fat and high carbs (LFD), to assess the complex interactions between stress, diet, and reward regulation. Our aim is to characterize deficits in weight gain, social interaction, reward, and antidepressant responses. We hypothesize that emotional stress, will accelerate the detrimental effects of WSDs, specifically a diet high in both fats and carbohydrates or HFD.

## Materials and Methods

### Animals

Male C57BL/6J mice (C57; Jackson Labs, Bar Harbor, Maine), postnatal day (PD) 28 at time of arrival, and CD-1 retired breeders (Charles Rivers, North Carolina) were housed at 23°C, in clear polypropylene boxes, on a 12-h light/dark cycle (lights on at 7 AM, lights off at 7 PM) and habituated for 7 days. During habituation, mice were handled briefly for several days prior to the start of the experiment. The C57 mice were group housed during habituation, and moved to single housing at PD35, the start of experimental manipulations, however, due to their highly aggressive nature, the CD-1 mice were singly housed upon arrival. Environmental enrichment was not given to any experimental mice to prevent interference with stress paradigm. Experimental procedures were conducted in strict compliance with the Guidelines for the Care and Use of Mammals in Neuroscience and Behavioral Research [National Research Council (US)] Committee on Guidelines for the Use of Animals in Neuroscience and Behavioral Research, 2003 (National Research Council, 2015) and approved by Texas A&M University’s Animal Care and Use Committee.

### Stress

Vicarious social defeat stress (VSDS) was performed as described previously (Sial et al., 2016). Importantly, for this defeat paradigm, CD-1 retired breeders are used as aggressors since they exhibit a heighted level of baseline aggression in response to novel intruder mice and therefore are single housed throughout the experiment. CD-1 mice are screened for 3 days prior to the start of the defeat to ensure stable and consistent levels of aggression. Adolescent mice were randomly assigned into control (CON), emotional (ES), or physical stress (PS) conditions and exposed to 10 days of VSDS lasting 10 min each. Briefly, the home cage of the CD-1 mouse was separated into two compartments by a perforated clear Plexiglas divider. CD-1 aggressors were housed on one side of a divided cage during the time of the defeat. The mice were housed in an open system as previously described (Golden et al., 2011; Sial et al., 2016) for the duration of the defeat. More specifically, mice were housed in clear rectangular hamster cages [26.7 cm (w) 48.3 cm (d) 15.2 cm (h)] while going through the defeat procedure. During the daily stress sessions, the intruder mouse (PS) was placed into the territorialized home-compartment of a CD-1 and subsequently physically defeated. At the same time, a different experimental mouse (ES) in the adjacent compartment witnessed the defeat bout, mimicking a form of psychological social stress. At the end of the defeat session, the ES-exposed mouse is moved to stay overnight in an empty compartment of an adjacent cage that was not used that day for defeats. ES-mice are never in physical contact with the CD-1 aggressor. The PS-exposed mouse was placed overnight in the compartment adjacent to the CD-1 that socially defeated it, for overnight sensory exposure. To minimize physical injury, the daily sessions were terminated when the intruder mouse adopted a persistent submissive posture, or the CD-1 displayed excessive physical aggression. Mice in the CON condition were housed on opposite sides of a Plexiglas divided cage and moved to an adjacent compartment daily. CON mice have with no contact with a CD-1. The mice were single housed in a closed system after the defeat 19.69 cm (w) × 30.48 cm (d) × 16.51 cm (h) and were housed in separate rooms.

### Diet

Mice were further randomized to receive either standard normal chow (NC; Teklad Rodent Diet; 8604; fat content 14% kcal, carbohydrate content 54% kcal from starches; 3.0 kcal/g), or a diet high in both fats and carbohydrates (HFD; Research Diets; D12451; fat content 45% kcal from lard, carbohydrate content 35% kcal from sucrose; 4.73 kcal/g) *ad libitum*. A separate diet that was low in fat but containing the same amount of added sucrose as the HFD was also utilized to isolate effects of fat (LFD; Research Diets; D1240K; fat content 10% kcal from lard, carbohydrate content 70% kcal from sucrose; 3.82 kcal/g). All food was placed in metal food hoppers and weighed daily. Caloric intake was measured by multiplying the daily consumption of food (g) by the caloric content (kcal/g) of the diet. The adjusted caloric intake is a normalized value that represents the number of calories consumed relative to body weight (g) of the mouse [(kcal/g of diet × grams of diet consumed)/body weight].

### Social Interaction Test

The social interaction test (SIT), a behavioral paradigm assessing social avoidance (Berton et al., 2006; Golden et al., 2011), is used as the primary behavioral outcome measure after exposure to the VSDS paradigm. The SIT was performed 24 h after the last defeat session, with an additional SIT performed following fluoxetine treatment. Briefly, the SIT is composed of two, 2.5 min-sessions. In the first session, a mouse is allowed to explore an open field arena (40 cm × 40 cm) containing an empty wire mesh cage (i.e., no social target present). For the second session, the mouse is then removed, and a novel CD-1 mouse is placed into the wire mesh cage (i.e., social target present). The test mouse is returned to the arena and the amount of time spent in the “interaction zone” (8 cm wide corridor surrounding the wire mesh cage), as well as the time spent in the corners farthest from the social target is measured. The VSDS-defeated mice explore the interaction zone significantly less when a target mouse is present and spend more time in the corners. Social interaction is calculated as a ratio of time spent in the interaction zone, with and without the target present (time in interaction zone with target/time in interaction zone without target). Interaction ratio below 1.0 indicate social avoidance and susceptibility to stress.

### Saccharin Preference Test

The saccharin preference test is a two-bottle choice paradigm (Hoffman, 2016) in which mice are given unrestricted access to both water and saccharin (Sigma-Aldrich: 240931). This paradigm has been used extensively to assess the effects of stress-induced anhedonia (Warren et al., 2011). Testing began 48 h after the last defeat session. Mice were habituated to two bottles for 2 days, and every 24 h the bottles were weighed, and their positions rotated (left to right and vice versa) to account for any side preferences. Mice were exposed to water and saccharin (0.05, 0.1, and 0.5%) for 48 h per concentration. The preference for saccharin over water [saccharin/(saccharin + water)] was used as a measure of sensitivity to reward. This formula takes total consumption into account and controls for differences in fluid intake.

### Fluoxetine Reversal

To determine potential changes in antidepressant efficacy after VSDS + WSD exposure, mice were given access to fluoxetine for 21 days. Fluoxetine hydrochloride (FLX; TCI America: F0750) was dissolved in the drinking water (80 mg/L) and made available *ad libitum*. The FLX dose was selected to achieve plasma levels close to 10 mg/kg (Dincheva et al., 2017). This approach was used to avoid potential stress-induced weight loss and/or tissue damage due to injection (Perrone et al., 2004). Bottles were covered in aluminum foil to prevent photodegradation and were measured daily to monitor fluid consumption. The FLX solution was replaced every 48 h to ensure purity and accurate concentration.

### Experiments Design

**FIGURE 1:**
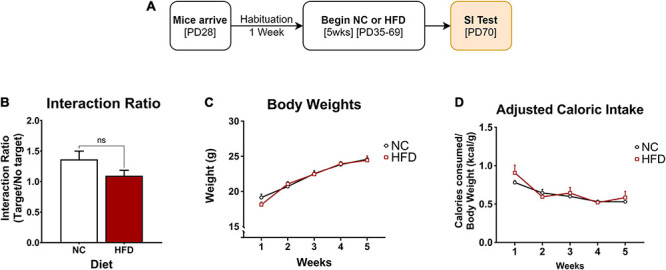
Effects of 5 weeks of HFD on body weight, adjusted caloric intake, and social interaction. **(A)** Adolescent mice were habituated for one week, then exposed to 5 weeks of NC (*n* = 8) or HFD (*n* = 8) and tested for social interaction. **(B)** No difference in social interaction between the NC- and the HFD-exposed mice (*p* > 0.05). **(C)** Mice in both NC and HFD conditions gained weight over time (main effect: *p* < 0.001) without an effect of diet (main effect: *p* > 0.05). **(D)** Each group showed a significant increase in the adjusted caloric consumption in week 1 compared to the rest of the weeks (*post hoc*: ^∗^*p* < 0.05: week 1 vs week 2), without significant differences between the two groups (*p* > 0.05). ^∗^*p* < 0.05: significantly different from NC. All data are expressed as the mean ± SEM.

A total of 106 mice were used in this study. In experiment 1 (Figures 1A–D) adolescent mice were exposed to 5 weeks of either normal chow (NC; *n* = 8) or high-fat/high-carbohydrate diet (HFD; *n* = 8). See groups breakdown below. An overview of experimental designs and results can be found in Table 1. At the end of the 5 weeks, the mice were exposed to social interaction test (SIT; Figure 1D).

**TABLE 1:** Breakdown of experimental design and the results have been summarized here.

**Cohort**	**Experimental design**	**Diet used**	**Stress used**	**Behavior**	**Results**
1	Exposure to 5 weeks of diet	Normal chow (NC) or High fat/high carbohydrate diet (HFD)	No stress	Social Interaction	No changes in body weight after 5 weeks of diet exposure during adolescence.
2	Exposure to VSDS before diet-exposure	Normal chow (NC) or High fat/high carbohydrate diet (HFD)	Vicarious social defeat stress	Social Interaction, Saccharin Preference Test	Adolescent exposure to psychological or physical stress leads to rapid weight gain and reduction in saccharin preference in response to high fat/high carbohydrate diet.
3	Exposure to VSDS before diet-exposure	Normal chow (NC), Low fat/high carbohydrate (LFD), or High fat/high carbohydrate diet (HFD)	Vicarious social defeat stress	Social Interaction, Fluoxetine Reversal	Exposure to low fat/high carbohydrate diet after stress does not lead to rapid weight gain, but similarly to high-fat diet, reduces fluoxetine efficacy.


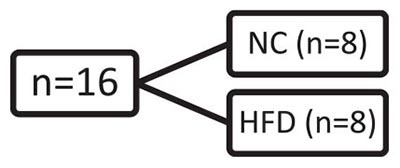


**FIGURE 2:**
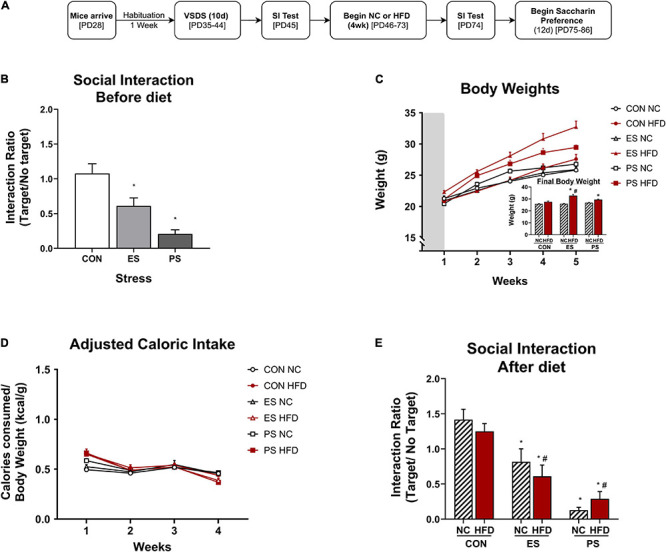
Effects of VSDS exposure prior to 4 weeks of HFD on social interaction, body weight, and caloric intake. **(A)** Adolescent mice were exposed to the vicarious social defeat stress (VSDS) paradigm and subsequently tested for social interaction. **(B)** Mice in both the emotional (ES) (*n* = 12, *p* < 0.001) and physical (PS) stress (*n* = 12, *p* < 0.05) condition showed a significant reduction in social interaction when compared to the CON-exposed mice (*n* = 12) with a main effect of stress (*p* < 0.001). **(C)** Mice that received HFD in both ES and PS conditions gained weight rapidly and were significantly heavier than the mice in the CON-NC condition during the final week of diet exposure (*post hoc*: *p* < 0.05) and a significant interaction between stress, diet and time (separated *n* = 6/group, *p* < 0.05). **(D)** No differences in adjusted caloric intake between any of the groups in the final week of diet exposure were noted (*post hoc, p* > 0.05), though there were significant differences in the first week (main effect of time and stress, *p* < 0.05). **(E)** To evaluate the effect of 4 weeks of HFD exposure on social interaction, the mice were exposed to the social interaction test (SIT) 24 h after the last day of diet exposure. Mice in the PS condition retained their defeated phenotype (i.e., social avoidance), as the PS + NC- (*p* < 0.01) and the PS + HFD-exposed (*p* < 0.01) were significantly different from the mice in the CON + NC and CON + HFD conditions with a main effect of stress (*p* < 0.05). Although the mice in the ES condition showed moderate increase in social interaction from baseline after 1 month of diet exposure, those in the ES + HFD condition still showed significant reduction in social interaction (*p* < 0.05) when compared to both the CON + NC- and the CON + HFD-exposed mice. *p < 0.05: significantly different than the CON-NC; ^#^*p* < 0.05: significantly different than CON-HFD. All data are expressed as the mean ± SEM.

**FIGURE 3:**
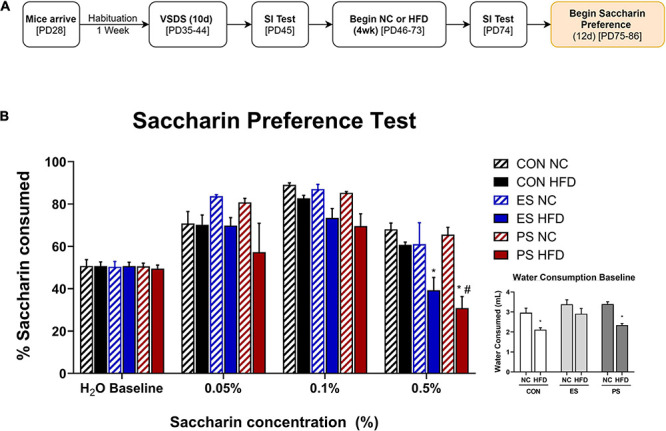
Effect of VSDS exposure followed by HFD on preference on saccharin. **(A)** Adolescent mice (*n* = 6/group) were used to further assess the effects of VSDS + HFD exposure on reward sensitivity using a two-bottle saccharin preference test. **(B)** There were no differences between any group in percentage of water consumed at baseline. In addition, no significant differences were observed at the 0.05 and 0.1% saccharin concentration. However, at the 0.5% concentration, both the ES + HFD- and PS + HFD-exposed mice consumed significantly less saccharin as compared to the CON + NC group (*post hoc*, *p* < 0.05). A MANOVA revealed differences in preference for the sweetened solution as a factor of saccharin concentration (*main effect; p* < 0.05); and diet (*main effect; p* < 0.05). Only the PS + HFD-exposed mice showed a significant difference when compared to the mice in the CON + HFD condition (*post hoc p* < 0.05). Inset graph shows the raw consumption during the water baseline. A two-way ANOVA revealed that the CON + HFD- and the PS + HFD-exposed mice drank less water than their NC-counterparts (*post hoc p* < 0.05). Mice in the ES + HFD condition trended toward a decrease, but this was not significantly different from the ES + NC-exposed mice (*post hoc p* > 0.05). Despite the HFD-induced adipsia, the relative percentage of saccharin consumed remained lower in the stressed group exposed to HFD. These findings indicate that concurrent stress and HFD exposure results in dysregulated sensitivity to reward (i.e., saccharin), independent of the caloric value of the sweetened solution. *p < 0.05: significantly different from CON + NC; ^#^*p* < 0.05: significantly different from CON + HFD.


In experiment 2 (Figures 2A–E, 3A-B), adolescent mice (*n* = 36) were first exposed to vicarious social defeat stress (VSDS; *n* = 12/group) and tested in the SIT 24 h after the last stress exposure (Figure 2B). 24 h after the SIT, the mice were randomly assigned to either NC or HFD (*n* = 6/group) for 4 weeks. See group breakdown below. At the end of the 4 weeks, another SIT (Figure 3B) was given. 24 h after this second SIT, the mice are exposed to saccharin preference testing.

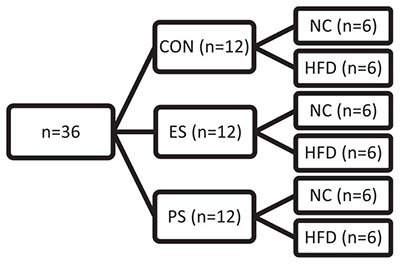


**FIGURE 4:**
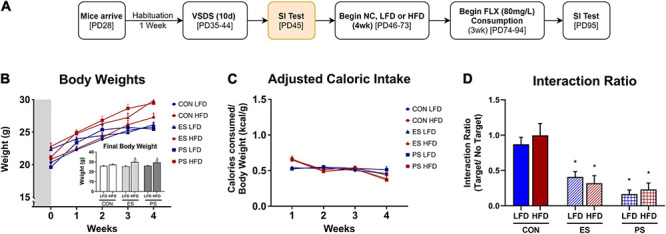
Effects of VSDS exposure prior to 4 weeks of LFD or HFD on social interaction, body weight, and caloric intake. **(A)** Adolescent mice underwent VSDS (*n* = 6/group) and subsequently tested for social interaction. **(B)** Mice in the ES + and PS + HFD conditions gained weight rapidly and were significantly heavier than the mice in the CON + LFD condition during the final week of diet exposure (*post hoc*; *p* < 0.05) with an interaction effect between stress, diet and time (*p* < 0.05). There was no difference in body weight between the CON + LFD- and the CON + HFD-exposed mice. **(C)** There were no differences in adjusted caloric intake between the groups in the final week of diet exposure (*post hoc p* > 0.05). Novelty induced hyperphagia in the first week results in a main effect of time (*p* < 0.05) and diet (*p* < 0.05). **(D)** Mice in the ES + LFD (*p* < 0.001) and the ES + HFD conditions (*post hoc p* < 0.05) showed an interaction ratio significantly lower than their CON-counterpart. The PS + LFD- and the PS + HFD-exposed mice also showed a significant reduction in social interaction compared to their respective CON-counterpart mice (*post hoc p* < 0.05, respectively). There was no difference between the interaction ratio of LFD and HFD exposed mice in either ES or PS groups. All data are expressed as the mean ± SEM. ^Δ^*p* < 0.05: significantly different than their respective LFD-exposed counterparts.

**FIGURE 5:**
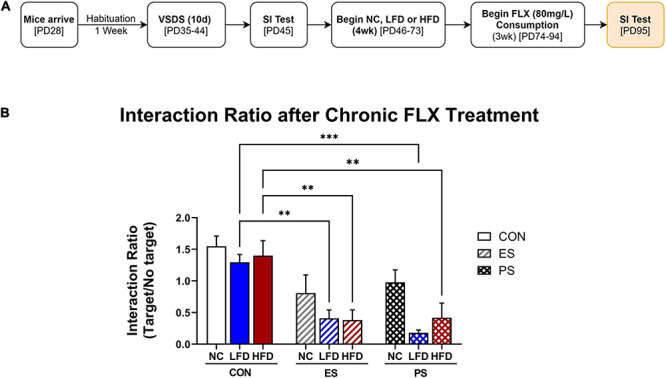
VSDS exposure before WSD during adolescence attenuates response to fluoxetine. **(A)** To assess the antidepressant efficacy of fluoxetine (FLX) after VSDS and WSD exposure, mice received FLX (80 mg/L) in the drinking water for 21 days (*n* = 5/group). **(B)** Mice in the ES condition that received either LFD or HFD showed an attenuated response to FLX, as the interaction ratio was significantly lower from the mice in the CON + NC condition. Similar effects of attenuated FLX response were seen for the mice in the PS condition regardless of diet exposure (main effect; diet *p* < 0.01 and stress *p* < 0.0001). Data are shown as mean ± SEM. ^∗^*p* < 0.05 **p < 0.01.


In experiment 3 (Figures 4A–D, 5A,B), adolescent mice were first exposed to VSDS *n* = 12/group) and tested in the SIT 24 h after the last stress exposure (Figure 3B). 24 h after the SIT, mice were randomly assigned to either NC (*n* = 5/group), LFD or HFD (*n* = 6/group) for 4 weeks. See groups breakdown below. At the end of the 4 weeks, another SIT (Figure 3B) was performed. 24 h after the second SIT, the mice were exposed to fluoxetine for 3 weeks and then receive a final SIT.

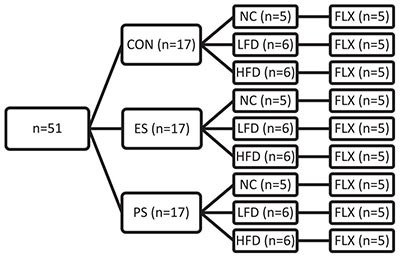


### Statistical Analyses

Data were analyzed using SPSS (version 26) and GraphPad Prism (version 9) software. Changes in body weight, food consumption and adjusted caloric intake were compared using multivariate analysis of variance (MANOVA), followed by Tukey HSD *post hoc* tests with stress (CON, ES, and PS), diet (NC, LFD, and HFD), and time (averaged 7 days across week: repeated measure) as sources of variance. When appropriate, two-way ANOVA or Student’s *t*-tests were used to determine statistical significance of pre-planned comparisons. Data are expressed as the mean ± SEM, with statistical significance set at *p* < 0.05.

## Results

### Effects of 5 Weeks of Diet on Body Weight, Caloric Intake, and Social Interaction

#### Experimental Design

After habituation, adolescent male C57 mice were moved from grouped to individual housing and randomly assigned to either the NC (*n* = 8) or HFD (*n* = 8) conditions to assess the effects of HFD on body weight, food consumption, adjusted caloric intake, and social interaction (SI). The mice were exposed to 5 weeks (PD35-69) of diet before measuring SI (Figure 1A).

#### Social Interaction

The effect of NC and HFD exposure on social interaction was examined and represented by an interaction ratio. An unpaired *t*-test revealed no significant differences between the groups (*t*_(__14__)_ = 1.672; *p* > 0.05), indicating that 5 weeks of HFD alone had no effect on social interaction (Figure 1B).

#### Body Weight

Body weight was measured daily and averaged across the week to yield weekly group averages (Figure 1C). A two-way repeated measures ANOVA showed that mice in both the NC and HFD conditions gained weight over time (*F*_(__2,39__)_ = 147.4; *p* < 0.0001) but did not differ from each other as a function of diet exposure (*F*_(__1,14__)_ = 0.09; *p* > 0.05). No differences in weight gain between groups were detected throughout the 5 weeks (*p* > 0.05).

#### Adjusted Caloric Intake

Weekly caloric intake was measured as described, then divided by their respective body weight and averaged across each week to depict the adjusted caloric intake (Figure 1D). Differences in adjusted caloric intake were a function of time (*F*_(__2,37__)_ = 11.79; *p* < 0.0001), and not diet (*F*_(1,14)_ = 0.97; *p* > 0.05). This difference is underlined by an adjusted caloric consumption in both groups in week 1 that is significantly higher as compared to the remaining four weeks (*p* < 0.05). Overall, these findings show that 5 weeks of HFD does not yield short-term (1-month) effects on caloric intake or body weight, except for the first week of diet exposure, an effect that may have been the result of novelty-induced hyperphagia in response to the novel diet.

### Effects of VSDS Exposure Followed by HFD on Body Weight, Caloric Intake, and Social Interaction

#### Experimental Design

After a week habituation period, adolescent mice were randomly assigned into CON and VSDS (ES or PS) conditions (*n* = 12/group) and exposed to VSDS for 10 days (PD35-44), followed by a SIT 24 h after the final defeat session. After VSDS exposure, the mice were randomized further into either NC or HFD conditions to assess the effects of VSDS + HFD on body weight, food consumption, and adjusted caloric intake (Figure 2A). Effects of VSDS + HFD on SI and saccharin preference are presented in Figures 3, 4, respectively.

#### Social Interaction Before Diet

To verify the efficacy of VSDS exposure, mice were assessed for social interaction as measured in the SIT. Interaction ratios were calculated as described. A two-way ANOVA revealed a significant main effect of stress (*F*_(__2,22__)_ = 12.90; *p* < 0.0002). *Post hoc* analysis showed that the interaction ratio of both ES- and PS-exposed mice were significantly lower (i.e., social avoidance) than the mice in the CON condition indicating a successful defeat (Figure 2B). Although the PS-exposed mice had lower SI scores, the magnitude did not significantly differ when compared to the ES-exposed group (*p* > 0.05).

#### Body Weight

Measurements of body weight were obtained daily, starting across the defeats and throughout the 4 weeks of diet exposure, and averaged weekly (Figure 2C). A 3 (stress) × 2 (diet) × 5 (time: weekly body weight as repeated measured variable) MANOVA revealed a significant three-way interaction between stress, diet and time (*F*_(__8,54__)_ = 2.23, *p* < 0.05; Wilks’ Λ = 0.565). *Post hoc* analyses revealed that at the final week of food consumption the ES + HFD and PS + HFD-exposed mice were significantly heavier than the CON + NC-exposed group (*p* < 0.0025 and *p* < 0.0007, respectively). Surprisingly, only the ES + HFD- differed significantly from the CON + HFD-exposed group (*p* < 0.01). No changes in body weight between the ES + HFD- and PS + HFD-exposed groups were detected (*p* > 0.05).

#### Adjusted Caloric Intake

Caloric intake was measured and converted to adjusted caloric intake and averaged across the 4 weeks of diet exposure (Figure 2D). A MANOVA revealed significant changes in adjusted caloric intake as a factor of time (*F*_(__3,27__)_ = 71.98; *p* < 0.05; Wilks’ Λ = 0.111) and diet (*F*_(__3,27__)_ = 8.798; *p* < 0.05; Wilks’ Λ = 0.506), but not stress (*F*_(__6,54__)_ = 1.29; *p* > 0.05; Wilks’ Λ = 0.774). *Post hoc* analysis detected significant differences in the first week between HFD- and NC-exposed mice without differences seen between the groups during the final week.

#### Social Interaction After Diet

To evaluate whether 4 weeks of HFD after VSDS would influence social interaction, mice underwent a SIT 24 h after the last day of diet exposure (Figure 2A). A two-way ANOVA indicated only a significant effect of stress (*F*_(__2,30__)_ = 35.91; *p* < 0.0001; Figure 2E). *Post hoc* analyses showed that mice in both the ES + HFD- (*p* < 0.02) and PS + HFD-exposed (*p* < 0.0002) conditions retained their social avoidance phenotype as they were significantly different from the CON + HFD-exposed group. More specifically, mice in the PS condition retained their defeated phenotype, as the PS + NC- and PS + HFD-exposed groups showed significantly more social avoidance when compared to the CON + NC control mice (*p* < 0.01, respectively). Although the mice in the ES + NC condition showed a moderate increase in social interaction from baseline after 1 month of diet exposure, the ES + HFD-exposed mice still showed a significant reduction in interaction (*p* < 0.05) when compared to the mice in both CON + NC and CON + HFD conditions.

### Effects of VSDS Exposure Followed by HFD on Saccharin Preference

To evaluate the effects of VSDS + HFD exposure on reward sensitivity, mice were assessed for preference to a sweetened water solution for 12 days (PD75-86) using a two-bottle preference test, 24 h after the last SIT (Figure 3A). To this end, we ran a two-bottle saccharin preference test with varying saccharin concentrations (Figure 3B). A MANOVA revealed differences in preference for the sweetened solution as a factor of saccharin concentration (*F*_(__3,28__)_ = 122.39; *p* < 0.05; Wilks’ Λ = 0.071) and diet (*F*_(__3,28__)_ = 7.11; *p* < 0.05; Wilks’ Λ = 0.568). *Post hoc* analyses revealed that there were no differences between the groups at the 0.05 and 0.1% concentrations. However, both the ES + HFD- and PS + HFD-exposed mice showed a significant reduction in preference for saccharin at the 0.5% when compared to the CON + NC-exposed group (*p* < 0.05). Furthermore, only the PS + HFD-exposed group was significantly different when compared to the CON + HFD-exposed mice (*p* < 0.05). Inset graph shows the raw water consumption during the water baseline. A two-way ANOVA showed that the CON + HFD- and the PS + HFD-exposed mice drank less water than their respective NC-exposed counterparts (*p* < 0.05).

### Effects of VSDS Exposure Followed by LFD or HFD on Body Weight, Caloric Intake, and Social Interaction

To further elucidate whether the observed effects thus far were due to HFD consumption, a separate control diet, similar in carbohydrate content but lower in fat (LFD), was introduced.

#### Experimental Design

After a week habituation, adolescent male mice were exposed to 10 days of VSDS (*n* = 6/group), followed by 4 weeks of either NC, LFD or HFD, and then followed by a SIT (Figure 4A). After the SIT, these mice were exposed to 21 days of fluoxetine in their drinking water followed by another SIT, results which are shown in Figure 5.

#### Body Weight

A MANOVA detected significant changes in body weight as a result of stress, diet and time interaction (*F*_(__8,54__)_ = 2.21, *p* < 0.05; Wilks’ Λ = 0.568; Figure 4B). *Post hoc* analysis show that ES- and PS-exposed mice in the HFD condition gained weight more rapidly and were significantly heavier when compared to the LFD-exposed mice regardless of stress condition during the final week of diet exposure (*p* < 0.05; inset graph). Additionally, ES + HFD-exposed mice were significantly heavier than the CON + HFD group (*p* < 0.05; inset graph).

#### Adjusted Food Intake

Three-way MANOVA showed that differences in adjusted caloric intake were a factor of time (*F*_(__3,28__)_ = 151.89, *p* < 0.05; Wilks’ Λ = 0.152) and diet (*F*_(__3,28__)_ = 32.39, *p* < 0.05; Wilks’ Λ = 0.224; Figure 4C). No significant differences were detected between the groups during the final week of diet exposure (*p* > 0.05).

#### Social Interaction

After 4 weeks of diet exposure, the mice were tested for SI. A two-way ANOVA detected significant changes in SI as a factor of stress (*F*_(__2,30__)_ = 26.78, *p* < 0.0001; Figure 4D) but not diet exposure (*F*_(__1,30__)_ = 0.155; *p* > 0.05). *Post hoc* analysis revealed that VSDS-exposed mice showed significant reduction in SI regardless of stress (ES or PS) or diet (LFD or HFD) conditions when compared to their respective CON counterparts (*p* < 0.05).

### Effects of Fluoxetine Treatment on VSDS+ LFD– or HFD-Induced Social Avoidance

Given increasing rates of treatment-resistant depression in the youth (Maalouf et al., 2011), we explored whether stress and WSD exposure would influence antidepressant efficacy. To assess this possibility, mice from the same cohort reported in Figure 5 were given fluoxetine (FLX; 80 mg/mL) for 21 days in the drinking water, after exposure to VSDS and either NC, LFD or HFD (Figure 5A). A two-way ANOVA revealed that differences in antidepressant efficacy were a factor of both stress (*F*_(__2,36__)_ = 22.39; *p* < 0.0001) and diet (*F*_(__2,36__)_ = 5.525; *p* < 0.0081). *Post hoc* analysis showed that FLX treatment rescued the social interaction ratio of the mice in the ES + NC and PS + NC conditions. Interestingly, LFD- and HFD-exposed mice showed an attenuated response to FLX regardless of stress condition when compared the CON + NC-exposed mice. More specifically, both the ES + LFD- and PS + LFD- as well as the ES + HFD- and PS + HFD-exposed mice showed significantly lower interaction ratio when compared to their respective CON + LFD (*p* < 0.05), and CON-HFD (*p* < 0.05) groups. These findings indicate that WSD attenuates the antidepressant efficacy of FLX.

## Discussion

There is a bidirectional relationship between neuropsychiatric disorders such as major depression and post-traumatic stress, and diet-related disorders such as metabolic syndrome (MetS) and cardiovascular disease (Harris and Barraclough, 1998; Thombs et al., 2006; Luppino et al., 2010; Pan et al., 2012; Marazziti et al., 2014), with stress as a major modulating factor in the development of obesity in human and animal models (De Vriendt et al., 2009; Karatsoreos et al., 2010; Goto et al., 2014; Tajik et al., 2014). Surprisingly, despite the prevalence and negative consequences of these, often comorbid, conditions, there is a lack of research delineating neurobiological interactions between stress, metabolic and mood disorders. This is especially during adolescence, a developmental stage distinguished by increased vulnerability to stress and when the onset of mood disorders and adoption of poor eating habits often emerge (Paus et al., 2008; O’Neil et al., 2014; Banfield et al., 2016). Here we show, for the first time, the combined detrimental effects of early-life stress and western-style diets (WSDs) on body weight, reward sensitivity, and antidepressant efficacy in adolescent mice.

In this study, we first sought to determine whether 5 weeks of *ad libitum* consumption of a WSD, high in both fats and carbohydrates (HFD), during the sensitive period of adolescence would be sufficient to induce changes in weight gain and caloric intake in male mice. Although adolescent mice in both the normal chow (NC) and the WSD condition gained weight over time, this did not differ between the groups across the 5 weeks. This lack of weight gain is a common feature seen in adult rodents, as it can take 8-20 weeks, sometimes longer, and consumption of a more calorically dense, extremely-high fat diet to see significant weight gain and other deficits (van der Heijden et al., 2015; Moreno-Fernández et al., 2018; Speakman, 2019). Despite no significant changes in weight gain between the groups, the mice in the WSD condition consumed significantly less food as compared to the NC controls across the 5 weeks, indicating that the WSD-exposed mice did homeostatically regulate and consume less of the calorically dense diet. This finding was corroborated by the lack of changes observed in caloric intake between the groups. At the end of the 5 weeks of diet exposure, these mice were tested for social interaction with no differences observed between the groups. Together, these findings indicate that 5 weeks of WSD exposure does not influence social interaction, body weight or caloric intake, except for the first week of diet exposure, an effect that may have been driven by novelty-induced hyperphagia in response to the mice being moved into single housing or exposed to the novel diet (Schipper et al., 2018, 2019).

Given the known detrimental effects of poor diet in children and adolescents (Weiss et al., 2004; Fryar et al., 2018; Nehus and Mitsnefes, 2019), and how early-life stress can induce metabolic abnormalities resulting in life-long adverse consequences (Farr et al., 2015; Gonzalez-Bulnes et al., 2016), we assessed the potential negative behavioral effects of exposure to vicarious social defeat stress (VSDS) and HFD. The VSDS paradigm is an ethologically relevant and robust stressor that allows for the uncoupling of emotional stress (ES) from physical stress (PS). This paradigm has demonstrated that simply witnessing repeated physical defeats evokes many of the same behavioral deficits (e.g., reduced social interaction, lower latency to immobility, anhedonia) as those that experience direct PS (Warren et al., 2011). Following 10 days of VSDS exposure, adolescent mice were exposed to 4 weeks of either NC or HFD. We found that both ES- and PS-exposed mice in the HFD conditioned rapidly gained weight (within 1 week after VSDS exposure) when compared to non-stressed control (CON) and the VSDS-exposed mice in the NC condition at the end of the 4 weeks. Interestingly, ES + HFD-, but not the PS + HFD- were significantly heavier than CON + HFD-exposed mice. The lack of differences between the PS + HFD- and the CON + HFD-exposed mice are demonstrated in adult studies where the mice in the PS + HFD condition weigh more than the mice in the CON + NC but not the CON + HFD condition (Chuang et al., 2010). Interestingly, the rapid weight gain presented here in adolescent mice is independent of food or caloric intake, as the HFD-exposed mice consumed less grams of the calorically dense diet, regardless of stress condition, and show no differences in adjusted caloric intake, despite novelty-induced hyperphagia observed following single housing and introduction to the novel diet. Stress is typically known to decrease food intake (Morley et al., 1983) and decrease weight gain (Warren et al., 2013; Ip et al., 2019), thus the nominal change in caloric intake in adolescents is intriguing, given their rapid increase in body weight. The mechanism(s) underlying this phenomenon in adolescents is unknown. In adult mice, increases in caloric intake of palatable diets is observed when first exposed to social stress (Coccurello et al., 2018; Hassan et al., 2019). Given that PS exposure can cause changes in lipid regulation and energy portioning (Chuang et al., 2010), it is possible that shifts in metabolic processing are occurring more drastically in adolescence after stress exposure, and therefore result in rapid weight gain despite consuming the same amount of calories as the mice in the NC condition. Further studies are needed to explore this hypothesis and determine whether altered cellular respiration pathways take place during adolescence to preferentially store and metabolize different sources of macronutrients.

Social avoidance is an integral symptom of depression and can be quantified in rodents using the social interaction test (SIT), a measure of stress reactivity and depressive/anxiety-like behaviors (Overstreet, 2012). An initial baseline measurement of avoidance was taken 24 h after the last VSDS exposure. As expected, both the ES- and PS-exposed mice showed significant reduction in social interaction compared to the mice in the CON condition. To evaluate whether HFD exposure influenced social interaction, mice were retested in the SIT 4 weeks after diet exposure. Interestingly, the PS + NC- and the PS + HFD-exposed mice retained their socially avoidant phenotype after diet consumption. Some studies have yielded inconsistent findings, showing that exposure to HFD before or during stress can buffer against the negative effects of PS (Finger et al., 2011; MacKay et al., 2017). The ES + HFD-exposed mice maintained comparable avoidant phenotypes as observed before diet exposure. These data show that 4 weeks of HFD has no effect on social interaction in either the ES- or PS-exposed mice.

The effect of HFD and stress on reward reactivity to saccharin, an artificial sweetener devoid of calories, was assessed using a two–bottle preference test, where the readout is given as a percentage of saccharin consumed over plain water. We report that mice exposed to either ES or PS showed decreased preference to 0.5% saccharin, a behavioral phenotype often associated with anhedonia (i.e., lack of pleasure), when compared to the CON + NC-exposed mice. HFD-stressed mice show less than 50% preference for saccharin, which could be interpreted as an aversion to the 0.5% saccharin concentration. There was a trend toward reduced consumption in HFD-stressed mice at the 0.1% concentration, however, this experiment needs to be repeated in a larger cohort in order to confirm our findings. Given the pivotal role played by the mesolimbic dopamine (DA) system in food consumption and reward (Wise, 2013; Volkow et al., 2017), it is conceivable that HFD and stress exposure compromise the DA reward system. The brain’s reward pathway, which includes the nucleus accumbens (NAc) and its dopaminergic input from the ventral tegmental area, are involved in regulating motivated behavior, along with responses to drugs of abuse and natural reward (Di Chiara and North, 1992; Kelley and Berridge, 2002; Bolaños and Nestler, 2004; Wallace et al., 2008). Experimental evidence indicates that exposure to sweet solutions such as sucrose are rewarding (Hajnal and Norgren, 2001; Datla et al., 2002) – they activate the mesolimbic DA system resulting in increases of DA release in the NAc, whereas lesions to this reward pathway block sucrose preference (Shimura et al., 2002). Reward dysregulation is a common phenotype that often emerges after early-life chronic exposure to palatable substances (Sato et al., 1991; Teegarden et al., 2009), drugs of abuse (Iñiguez et al., 2009; Naneix et al., 2017), or stress (Iñiguez et al., 2010; Parise et al., 2013). Chronic consumption of diets high in fats and carbohydrates have also been shown to also induce long term changes in DA neurotransmission (Teegarden et al., 2009; Naneix et al., 2018), thus influencing reward sensitivity. Exposure to VSDS decreases sucrose preference in both adults (Krishnan et al., 2007; Warren et al., 2013) and adolescent mice (Iñiguez et al., 2014), deficits associated with reduced reward sensitivity and anhedonia-like behavior in rodents (Eagle et al., 2016). Our results are in agreement with these findings and extend them to demonstrate that HFD-exposure in conjunction with early-life stress induces deficits in reward sensitivity compared to stress alone. It must be noted that the CON + HFD-exposed mice consumed less water at baseline than the mice in the CON + NC condition, a phenomenon also observed in the PS-exposed mice; while the ES + HFD-exposed mice showed a non-significant slight decrease in water intake. The mechanism(s) underlying this effect is unknown. Though HFD influenced fluid intake, these effects are controlled for by taking a percentage of saccharin consumed over total intake.

Although there is a substantial amount of evidence for the long-term detriments induced by western-style diet consumption (Zemdegs et al., 2016), the specific macronutrient composition that may directly or indirectly cause these deficits have not been clearly delineated. Traditionally, high-fat diets have taken the burden for causing diet-related disorders such as obesity and MetS, though recently, the role of carbohydrates has come under scrutiny for their involvement in these comorbidities (Kearns et al., 2018). To assess how HFD exposure differed from a diet that had low fat, but the same carbohydrate content (LFD; 35% kcal from sucrose), adolescent mice were exposed to VSDS before 4 weeks of either HFD or LFD -exposure. Though mice exposed to either diet consumed the same amounts of adjusted calories, surprisingly, only the ES + HFD- and PS + HFD-exposed mice rapidly gained weight as demonstrated previously (see Figure 2C). This indicate that high carbohydrates were not enough to induce rapid weight gain in the stress-exposed mice, and a combination of high fat and carbs were needed to induce these physiological effects. In addition, there was no difference in social interaction between the HFD- and LFD-exposed mice regardless of stress condition as they retained their avoidant phenotype. Given that chronic antidepressant treatment can reverse social avoidance after VSDS exposure (Warren et al., 2013) we tested whether the 21-day exposure to the selective serotonin reuptake inhibitor fluoxetine (FLX; 80 mg/L in the drinking water) could reverse the behavior deficits. When tested in the SIT 24 h after the last day of drug consumption, ES + NC and PS + NC mice treated with FLX were not statistically different from the mice in the CON condition. Conversely, exposure to LFD or HFD blocked FLX’s ability to reverse the social avoidant behavior regardless of stress condition. We thus show here that although mice exposed to stress do not gain weight as rapidly when consuming LFD, they share similar behavioral deficits as the HFD-exposed mice. Resistance to FLX treatment has previously been shown in adult mice subjected to two separate 7-week periods of unpredictable chronic mild stress while on a HFD (Isingrini et al., 2010). The mechanism(s) mediating this phenomenon is not known. Although speculative, reduced FLX efficacy may be due to over-sequestration of the lipophilic FLX into adipose stores (Shekar et al., 2012), or due to WSD-induced inflammation (Wu et al., 2018). The latter seems to be supported by studies showing that anti-inflammatory agents such as acetylsalicylic acid, when given with FLX, increase FLX’s antidepressant efficacy in animal models (Wang et al., 2011; Yang et al., 2014; Zdanowicz et al., 2017). There is a strong association between MetS and mood disorders, especially depression (Hiles et al., 2016; Penninx and Lange, 2018; Chan et al., 2019). Depending on the remission criteria utilized, the rate of treatment-resistant depression (TRD) ranges between 35 and 60% (Nemeroff, 2007; Fekadu et al., 2009), with TRD associated with higher rates of cardiovascular mortality (Carney and Freedland, 2009). Interestingly, diet-related disorders (e.g., MetS) contribute significantly to the sustained chronicity of depression and low rates of antidepressant efficacy (Chokka et al., 2006; van Reedt Dortland et al., 2010; Vogelzangs et al., 2011, 2014). These findings therefore indicate that diet plays a pivotal role in antidepressant efficacy and highlight the importance of considering diet when prescribing antidepressants.

A limitation of our study is that the findings reported were derived using male subjects. Reports shown that women are more likely to suffer from depression and are at higher risk of developing obesity after a stressful event (Mannan et al., 2016). The stress paradigm utilized in this study made it nearly impossible to use females, as successfully encouraging males to aggressively charge female mice is an ongoing obstacle in the field in creating a paradigm where females are exposed to physical stress. Although some approaches have included optogenetic manipulations in transgenic mice, using more aggressive strains of female mice, and the use of male pheromones to incite males to show aggression toward a female (Takahashi et al., 2017; Harris et al., 2018; Newman et al., 2019), these new models have not been fully characterized, and will require comprehensive testing to assess their true efficacy in adolescent rodents. Another limitation is the lack of a micronutrient- and protein-controlled chow. The normal chow used in our study is what is commonly given to rodents in many laboratories across the country, though using a matched control chow would have excluded the possibility of off-target effects. Given the reduction in fluid intake in HFD-mice, it is plausible that reduction in the efficacy of fluoxetine is related to reduced consumption. Future studies will need to utilize intraperitoneal injections to rule out the effect of adipsia. Furthermore, studies assessing physiological measures such as corticosterone, insulin and inflammatory markers would be critical in validating the paradigm in modeling adolescent metabolic-mood syndrome. Lastly, the data represented for body weight and caloric consumptions are depicted as absolute values, and the percent change from baseline can be found in the Supplementary Material.

Together, these findings indicate that WSD exposure during adolescence leads to physiological and reward-related deficits (i.e., anhedonia) that may lead to development of maladaptive behaviors and negative health outcomes in adulthood. We describe a paradigm that can elicit a rapid obesogenic-like phenotype, along with deficits in reward and antidepressant response. Elucidating the relationship between MDD and MetS may uncover crucial implications for adolescent health and sociocultural patterns of behavior for future adult functioning. Further, it is crucial to understand the neurobiological interactions between mood and metabolic disorders, as environmental insults such as social stress and unhealthy diet can cause long-lasting behavioral and physiological deficits that persist into adulthood (Lobstein et al., 2004).

## Data Availability Statement

The original contributions presented in the study are publicly available. This data can be found here: https://www.dropbox.com/s/qezm5os36z5lfmp/VSDS%20%2B%20WSD%20Manuscript%20Data%20Combined%20Prism%20-%20Sial%20et%20al.%2C%202021.pzfx?dl=0.

## Ethics Statement

The animal study was reviewed and approved by Texas A&M University IACUC.

## Author Contributions

OS designed and executed majority of the experiments. TG, AC-A, EV, and EC assisted with data collection and analysis. LP assisted with experimental design and data analysis. CB-G assisted with experimental design. All authors contributed to the article and approved the submitted version.

## Conflict of Interest

The authors declare that the research was conducted in the absence of any commercial or financial relationships that could be construed as a potential conflict of interest.

## Publisher’s Note

All claims expressed in this article are solely those of the authors and do not necessarily represent those of their affiliated organizations, or those of the publisher, the editors and the reviewers. Any product that may be evaluated in this article, or claim that may be made by its manufacturer, is not guaranteed or endorsed by the publisher.

## Supplementary Material

Supplementary Figure 1 Effects of 5 weeks of diet on % change in body weight and caloric intake. **(A)** Adolescent mice were habituated for one week, then exposed to 5 weeks of NC (*n* = 8) or HFD (*n* = 8) and tested for social interaction. **(B)** Changes in body weight were calculated as % change from baseline. There was a significant difference between NC- and HFD- exposed mice as a factor of time (F_(2__.668__, 37__.35__)_ = 113.9, *p* < 0.0001) and diet (F_(1,14)_ = 6.158, *p* < 0.05). **(C)** These changes in body weight are likely due to the increased consumption of the novel diet, as there are no differences in % change from baseline during the final week of weight measurement. **(D)** Adjusted caloric intake was converted to % change from baseline. We see a decrease in caloric intake in both groups as a factor of time (F_(2__.487,34__.82__)_ = 16.80, *p* < 0.0001).

Supplementary Figure 2 Effects of VSDS exposure followed by HFD on % change in body weight and caloric intake. **(A)** Adolescent mice were exposed to the vicarious social defeat stress (VSDS) paradigm and subsequently tested for social interaction. **(B)** There were significant differences in the percent change in body weight from baseline. Repeated measures MANOVA showed significant effect of time × stress (F_(8,52)_ = 2.405; Wilks’ Λ = 0.533), *p* < 0.001 and time × diet (F_(__4,26__)_ = 4.866; Wilks’ Λ = 0.572), *p* < 0.05 and an interaction of time × diet × stress (F_(8,52)_ = 2.67 Wilks’ Λ = 0.502), *p* < 0.05. **(C)** Weight change from baseline during the last week shows significant differences as a factor of stress (F_(2, 30)_ = 13.74; *p* < 0.05), diet (F_(1, 30)_ = 48.92, *p* < 0.05) and an interaction effect (F_(2, 30)_ = 6.888, *p* < 0.05). *Post hoc* analysis showed that there were differences between the ES-HFD and PS-HFD groups from CON-HFD *p* < 0.05. **(D)** There were significant differences in the percent change in adjusted caloric intake from baseline. Repeated measures MANOVA showed significant effect of time × diet (F_(3,27)_ = 2.985; Wilks’ Λ = 0.751), *p* < 0.05.

Supplementary Figure 3 Effects of VSDS exposure followed by LFD or HFD on % change in body weight and caloric intake. **(A)** Adolescent mice underwent VSDS and subsequently tested for social interaction. **(B)** There were significant differences in the percent change in body weight from baseline. Repeated measures MANOVA showed significant effect of time × stress (F_(8,52)_ = 7.231; Wilks’ Λ = 0.224), *p* < 0.001 and time^∗^diet (F_(4,26_ = 3.066; Wilks’ Λ = 0.679, *p* < 0.05) and an interaction of time × diet × stress (F_(8,52)_ = 2.347; Wilks’ Λ = 0.54, *p* < 0.05). **(C)** Weight change from baseline during the last week shows significant differences as a factor of stress (F_(2, 30)_ = 10.92; *p* < 0.05), diet (F_(1, 30)_ = 24.23; *p* < 0.05). **(D)** There were significant differences in the percent change in adjusted caloric intake from baseline. Repeated measures MANOVA showed significant effect of time × diet (F_(3,27)_ = 6.302; Wilks’ Λ = 0.588; *p* < 0.05).

Supplementary Figure 4 Estimated FLX consumption after VSDS and HFD exposure during adolescence. Consumption of fluoxetine (FLX) was estimated based on water consumption during the saccharin preference test baseline (Figure 3B). FLX intake was influenced by both diet (F_(1, 102)_ = 27.69; *p* < 0.0001) and stress (F_(2, 102)_ = 5.47l; *p* < 0.05). Post hoc analysis revealed that CON + HFD and PS + HFD consumed less FLX compared to their respective NC-exposed counterparts. Neither ES + HFD nor PS + HFD were significantly different from CON + NC mice.

## Abbreviations

C57: C57BL/6J
CSDS: Chronic social defeat stress
CVD: Cardiovascular disease
FLX: Fluoxetine
HFD: High fat/high carbohydrate diet
MetS: Metabolic Syndrome
NC: Normal chow
LFD: Low fat/high carbohydrate diet
PD: Postnatal day
SIT: Social interaction test
TRD: Treatment-Resistant Depression
VSDS: Vicarious social defeat stress
WSD: Western-style diet.
