# Supplementary figures and images for: Exposure to Vicarious Social Defeat Stress and Western-Style Diets During Adolescence Leads to Physiological Dysregulation, Decreases in Reward Sensitivity, and Reduced Antidepressant Efficacy in Adulthood
Source: Front Neurosci. 2021 Aug 2;15:701919. doi: 10.3389/fnins.2021.701919 (PMC8366028; doi:10.3389/fnins.2021.701919)

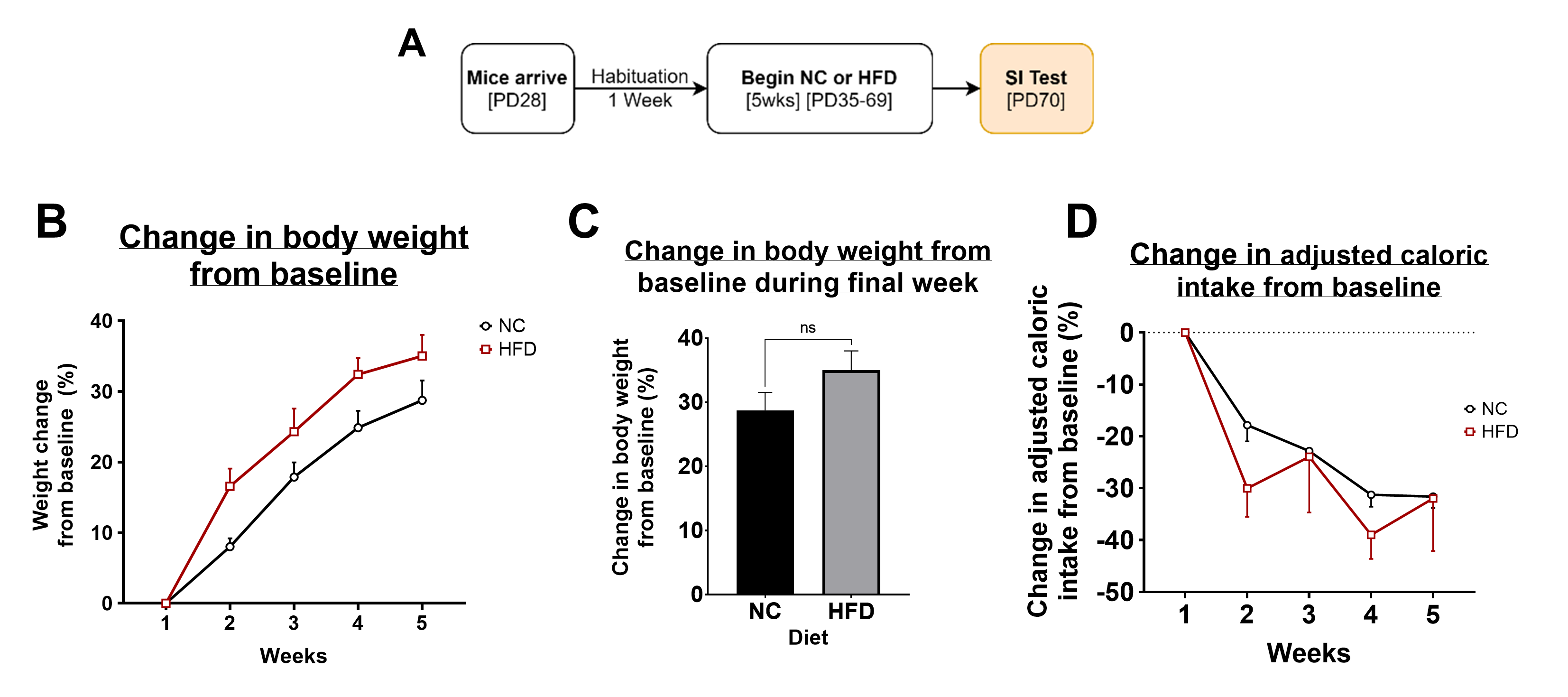

Supplement: Supplementary Figure 1 — Effects of 5 weeks of diet on % change in body weight and caloric intake. (A) Adolescent mice were habituated for one week, then exposed to 5 weeks of NC (n = 8) or HFD (n = 8) and tested for social interaction. (B) Changes in body weight were calculated as % change from baseline. There was a significant difference between NC- and HFD- exposed mice as a factor of time (F(2.668, 37.35) = 113.9, p < 0.0001) and diet (F(1,14) = 6.158, p < 0.05). (C) These changes in body weight are likely due to the increased consumption of the novel diet, as there are no differences in % change from baseline during the final week of weight measurement. (D) Adjusted caloric intake was converted to % change from baseline. We see a decrease in caloric intake in both groups as a factor of time (F(2.487,34.82) = 16.80, p < 0.0001). [file Image_1.TIF]

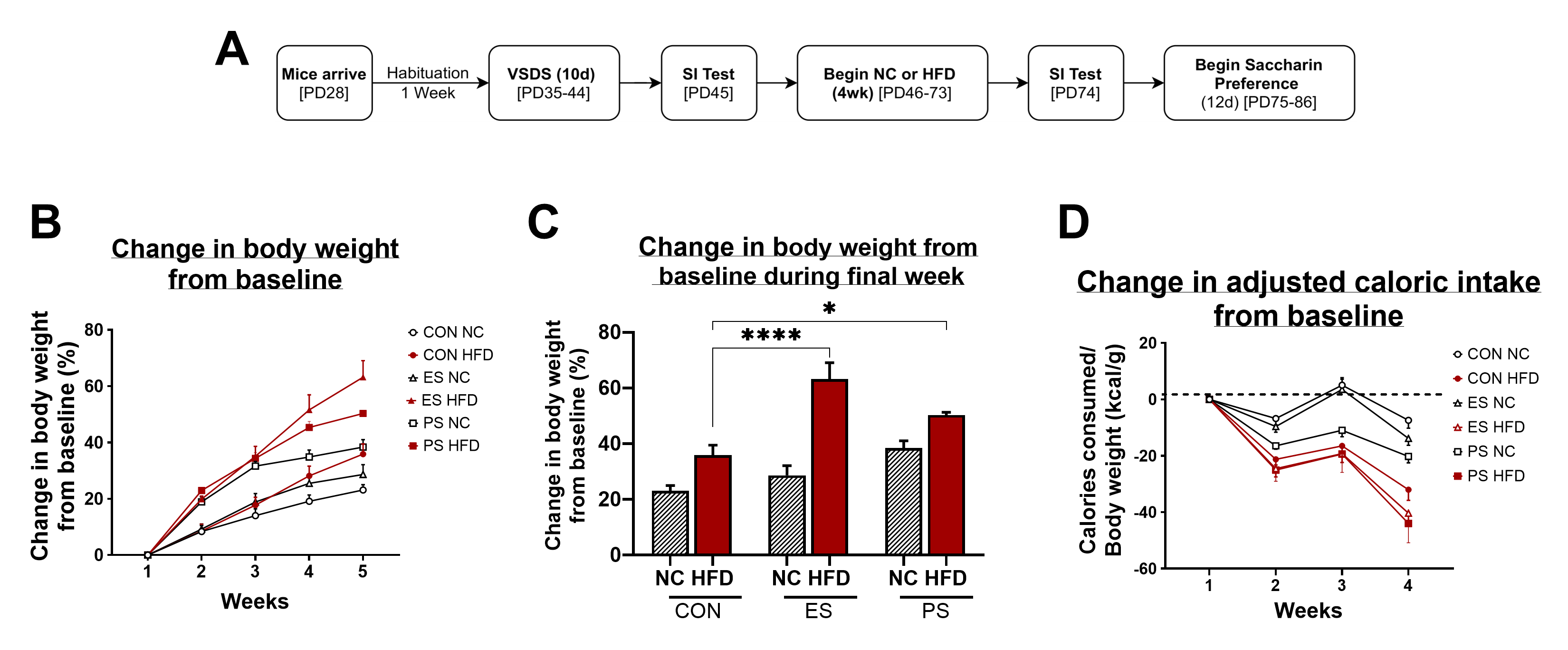

Supplement: Supplementary Figure 2 — Effects of VSDS exposure followed by HFD on % change in body weight and caloric intake. (A) Adolescent mice were exposed to the vicarious social defeat stress (VSDS) paradigm and subsequently tested for social interaction. (B) There were significant differences in the percent change in body weight from baseline. Repeated measures MANOVA showed significant effect of time × stress (F(8,52) = 2.405; Wilks’ Λ = 0.533), p < 0.001 and time × diet (F(4,26) = 4.866; Wilks’ Λ = 0.572), p < 0.05 and an interaction of time × diet × stress (F(8,52) = 2.67 Wilks’ Λ = 0.502), p < 0.05. (C) Weight change from baseline during the last week shows significant differences as a factor of stress (F(2, 30) = 13.74; p < 0.05), diet (F(1, 30) = 48.92, p < 0.05) and an interaction effect (F(2, 30) = 6.888, p < 0.05). Post hoc analysis showed that there were differences between the ES-HFD and PS-HFD groups from CON-HFD p < 0.05. (D) There were significant differences in the percent change in adjusted caloric intake from baseline. Repeated measures MANOVA showed significant effect of time × diet (F(3,27) = 2.985; Wilks’ Λ = 0.751), p < 0.05. [file Image_2.TIF]

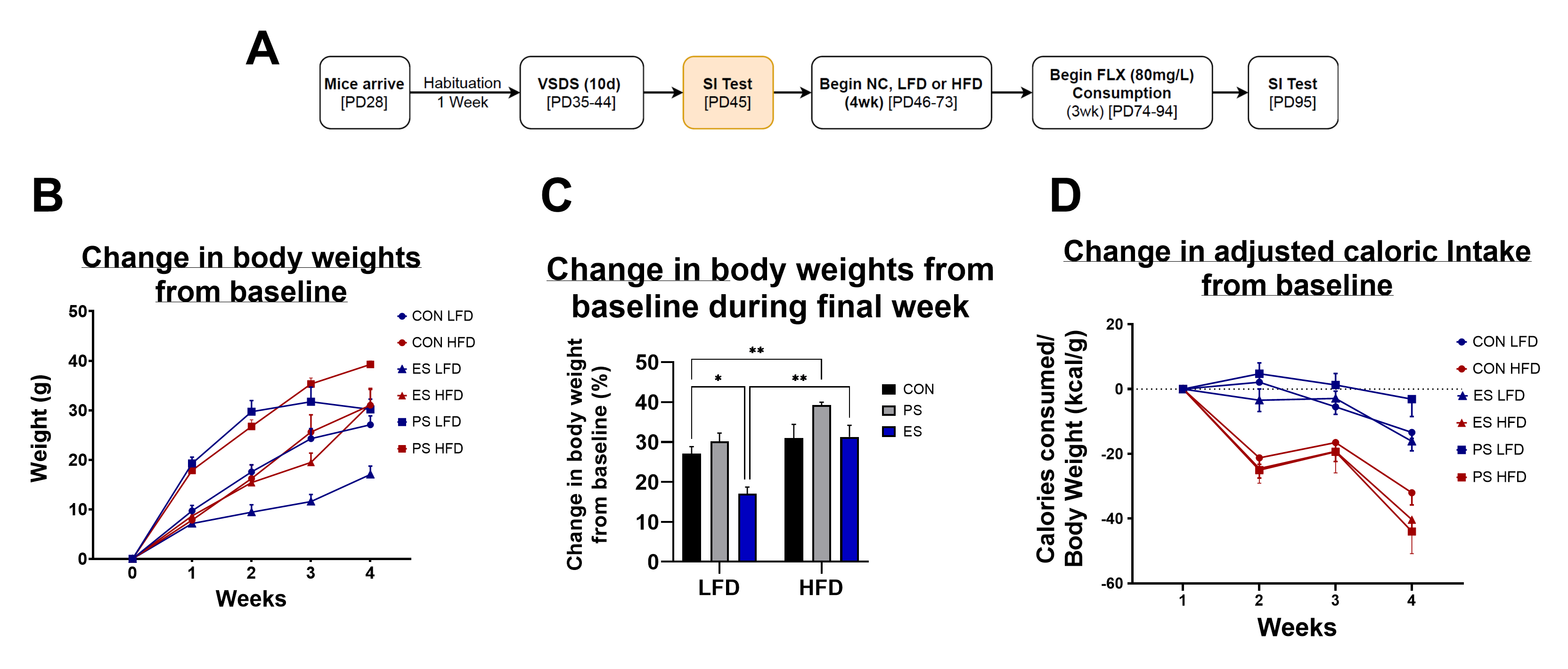

Supplement: Supplementary Figure 3 — Effects of VSDS exposure followed by LFD or HFD on % change in body weight and caloric intake. (A) Adolescent mice underwent VSDS and subsequently tested for social interaction. (B) There were significant differences in the percent change in body weight from baseline. Repeated measures MANOVA showed significant effect of time × stress (F(8,52) = 7.231; Wilks’ Λ = 0.224), p < 0.001 and time∗diet (F(4,26 = 3.066; Wilks’ Λ = 0.679, p < 0.05) and an interaction of time × diet × stress (F(8,52) = 2.347; Wilks’ Λ = 0.54, p < 0.05). (C) Weight change from baseline during the last week shows significant differences as a factor of stress (F(2, 30) = 10.92; p < 0.05), diet (F(1, 30) = 24.23; p < 0.05). (D) There were significant differences in the percent change in adjusted caloric intake from baseline. Repeated measures MANOVA showed significant effect of time × diet (F(3,27) = 6.302; Wilks’ Λ = 0.588; p < 0.05). [file Image_3.TIF]

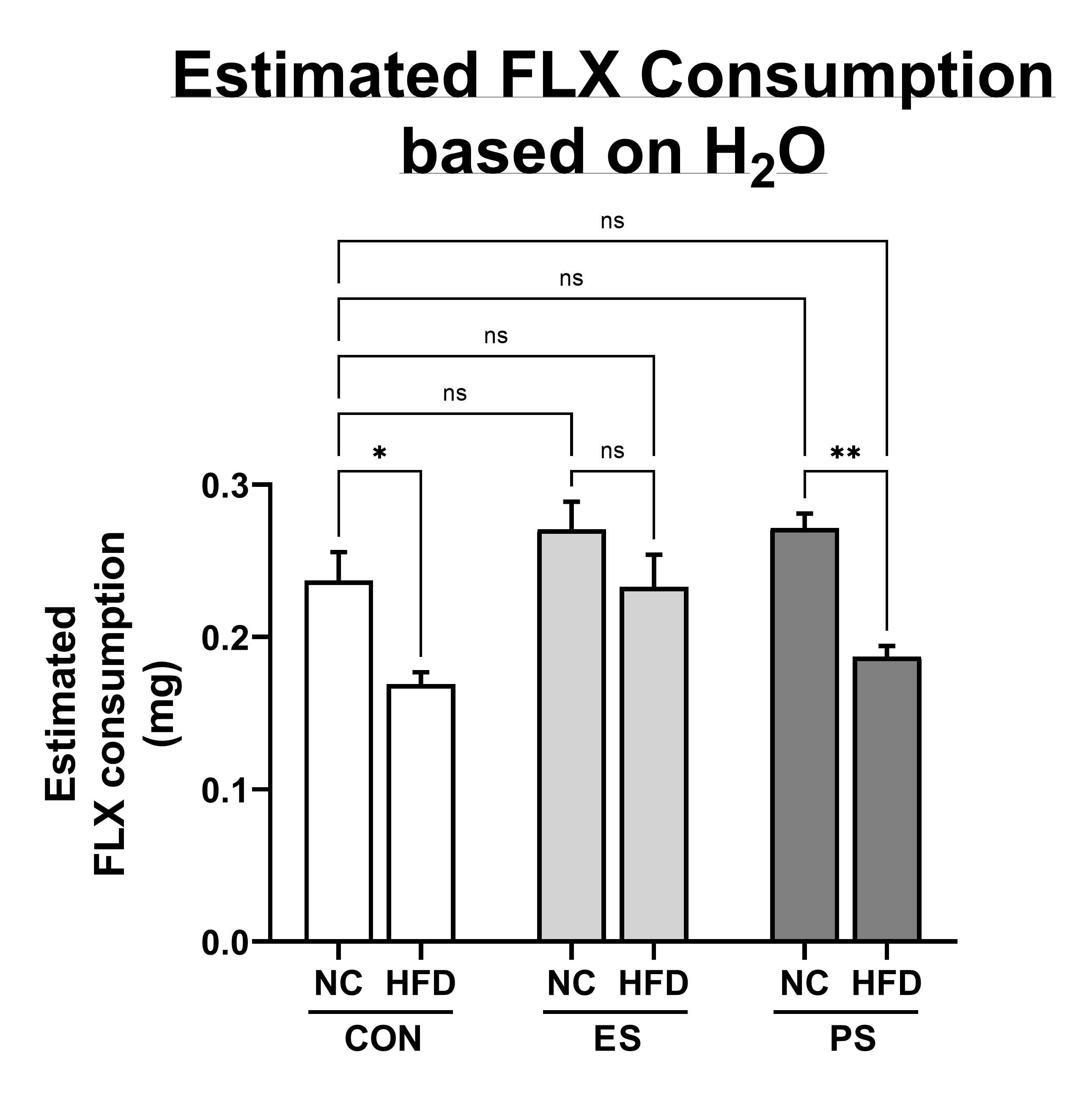

Supplement: Supplementary Figure 4 — Estimated FLX consumption after VSDS and HFD exposure during adolescence. Consumption of fluoxetine (FLX) was estimated based on water consumption during the saccharin preference test baseline (Figure 3B). FLX intake was influenced by both diet (F(1, 102) = 27.69; p < 0.0001) and stress (F(2, 102) = 5.47l; p < 0.05). Post hoc analysis revealed that CON + HFD and PS + HFD consumed less FLX compared to their respective NC-exposed counterparts. Neither ES + HFD nor PS + HFD were significantly different from CON + NC mice. [file Image_4.TIF]
